# Relationship of leukaemias with long-term ambient air pollution exposures in the adult Danish population

**DOI:** 10.1038/s41416-020-01058-2

**Published:** 2020-09-17

**Authors:** Robin C. Puett, Aslak Harbo Poulsen, Tahir Taj, Matthias Ketzel, Camilla Geels, Jørgen Brandt, Jesper H. Christensen, Mette Sørensen, Nina Roswall, Ulla Hvidtfeldt, Ole Raaschou-Nielsen

**Affiliations:** 1 Institute for Applied Environmental Health, School of Public Health, University of Maryland, College Park, MD USA; 2 Danish Cancer Society Research Center, Strandboulevarden 49, 2100 Copenhagen, Denmark; 3 Department of Environmental Science, Aarhus University, Frederiksborgvej 399, P.O. Box. 358, DK-4000 Roskilde, Denmark; 4 Global Centre for Clean Air Research (GCARE), University of Surrey, Guildford, GU2 7XH UK; 5 Department of Natural Science and Environment, Roskilde University, Roskilde, Denmark

**Keywords:** Risk factors, Leukaemia

## Abstract

**Background:**

Few population-based epidemiological studies of adults have examined the relationship between air pollution and leukaemias.

**Methods:**

Using Danish National Cancer Registry data and Danish DEHM-UBM-AirGIS system-modelled air pollution exposures, we examined whether particulate matter (PM_2.5_), black carbon (BC), nitrogen dioxide (NO_2_) and ozone (O_3_) averaged over 1, 5 or 10 years were associated with adult leukaemia in general or by subtype. In all, 14,986 adult cases diagnosed 1989–2014 and 51,624 age, sex and time-matched controls were included. Separate conditional logistic regression models, adjusted for socio-demographic factors, assessed exposure to each pollutant with leukaemias.

**Results:**

Fully adjusted models showed a higher risk of leukaemia with higher 1-, 5- and 10-year-average exposures to PM_2.5_ prior to diagnosis (e.g. OR per 10 µg/m^3^ for 10-year average: 1.17, 95% CI: 1.03, 1.32), and a positive relationship with 1-year average BC. Results were driven by participants 70 years and older (OR per 10 µg/m^3^ for 10-year average: 1.35, 95% CI: 1.15–1.58). Null findings for younger participants. Higher 1-year average PM_2.5_ exposures were associated with higher risks for acute myeloid and chronic lymphoblastic leukaemia.

**Conclusion:**

Among older adults, higher risk for leukaemia was associated with higher residential PM_2.5_ concentrations averaged over 1, 5 and 10 years prior to diagnosis.

## Background

Leukaemia, a cancer of the bone marrow, consists of a number of heterogeneous subtypes of haematological cancers. Leukaemia was the 13th most common diagnosed cancer worldwide in 2018.^1^ The age-standardised incidence rate per 100,000 persons per year for Denmark from 2012 to 2016 was 10.7 for males and 7.2 for females. These rates have remained fairly stable over the past 10 years.^2–4^ Leukaemia can be acute or chronic and of two main subtypes: lymphoblastic (or lymphocytic: lymphocyte over production) or myelogenous (or myeloid: granulocyte over production).

Little is known about risk factors for adult leukaemia, however, some research has shown higher risk associated with family history of hemopoietic cancers, and few lifestyle, environmental and demographic factors. Among environmental factors, benzene, ionising radiation and pesticide exposure have been explored. Workers exposed to benzene have shown higher leukaemia incidence.^5,6^ Higher rates of leukaemia have also been observed among Japanese atomic bomb survivors and Ukrainian Chernobyl clean-up workers.^7,8^ Studies of agricultural workers and pesticide applicators have also shown elevated risks among certain subpopulations.^9,10^

In limited research, air pollution has also been considered a potential risk factor, with most studies addressing childhood leukaemia. Results have been inconsistent, potentially due in part to differences in exposure assessment, study populations, and small sample sizes. An IARC/WHO 2016 review of childhood leukaemia and air pollutant exposures considered the evidence suggestive but inconsistent.^11^ A recent review found more convincing evidence for benzene than other air pollutant exposures.^12^ Among adults, no associations were observed in a Canadian population-based case-control study for all leukaemias with long-term particulate matter <2.5 microns in diameter (PM_2.5_) exposures.^13^ A Danish population-based case-control study showed increases in long-term nitrogen dioxide (NO_2_) and nitrogen oxides (NO_x_) exposures with increased odds for acute myeloid leukaemia but not for chronic myeloid or lymphoblastic leukaemia.^14^

To add information regarding pollutant exposures not previously examined and to investigate further the relationship of PM and NO_2_ with leukaemia incidence, we assessed the relationship of adult leukaemia diagnoses with long-term exposures to PM_2.5_, NO_2_, ozone (O_3_), and black carbon (BC), in a nationwide, Danish case-control study.

## Methods

### Population

Since 1968, all citizens of Denmark are assigned a personal identification number which allows access to healthcare and which can be used to link data from national health outcome registries with other registries containing information on potential confounders.^15,16^ For the current study, we took advantage of the Danish Cancer Registry, which tracks all cancers from 1943.^16,17^ We identified all leukaemia cases (International Classification of Diseases (ICD) 10 codes 90–95) in Denmark occurring between 1989 and 2014. We further categorised the leukaemias by subtype: acute lymphoblastic leukaemia (ALL, ICD10: 91.0), chronic lymphoblastic leukaemia (CLL, ICD10: 91.1), acute myeloblastic leukaemia (AML, ICD10 92.0) and chronic myeloid leukaemia (CML, ICD10: 92.1). We restricted to participants 20 years of age or older (Table S1). We excluded cases with prior cancer diagnoses (except non-melanoma skin cancer). Using the Danish Civil Registration System,^15^ for each case, we selected at random four controls, matched on sex, and birth month and year, who did not have a prior cancer diagnosis (except non-melanoma skin cancer) and were alive and had a Danish residential address at the date of leukaemia diagnosis for the case (index date). We further excluded cases and controls who: did not reside in Denmark at index date, were missing a geocodable residential address for 20% or more of the ten years prior to the index date, and those missing information on the covariates of interest. In main analyses, for the most conservative analysis, we excluded all topography codes other than C42.1, indicating bone marrow as the site of origin, according to ICD-O-3, IARC and NIH SEER rules.^18,19^ We also excluded cases and controls of non-Danish origin given the small proportion of immigrants and potential residual confounding from historical risk factors (e.g. other environmental exposures). We further excluded cases that were missing all controls and vice versa due to the earlier exclusions.

### Covariates

Information on smoking status was unavailable; however using data from Statistics Denmark, we were able to attach individual-level socio-economic position information: occupation, marital status and calendar year-specific disposable income (standardised for inflation); and area-level socio-economic position variables indicating the percentage of population at the parish level: with basic education as the highest education level attained, with income in the lowest quartile, owning their home, and retired. Individual and parish level variables where assessed 1 year before index date. Socioeconomic position individual and area-level variables were selected for parsimonious modelling and based on prior research,^13,14^ including use of the Danish Cancer Registry to examine the association of social inequality with leukaemia.^20^

### Exposures

The DEHM-UBM-AirGIS system models Danish population exposure to air pollutants and was designed and is continuously further developed by Aarhus University. The system and its validation are described in detail elsewhere.^21–29^ Briefly, the DEHM-UBM-AirGIS is a multi-scale coupled air pollution modelling system operating at high spatial (individual address) and temporal (hourly basis) resolution. The transport, dispersion and chemical transformation of air pollution is modelled as contributions from (1) regional background e.g. long-range transport from other countries, using the Danish Eulerian Hemispheric Model (DEHM)^25^; (2) national scale and urban background, taking into account the emission density originating from all types of emissions (e.g. traffic, industry and residential heating)^30,31^ and average building cover and height on a resolution of 1 km × 1 km, using the Urban Background Model (UBM)^21–24^; and (3) local street traffic, using information about the intensity, speed, type, and emission factors for the car fleet, street and building geometry, and meteorology, using the Operational Street Pollution Model (OSPM).^32^ The DEHM-UBM-AirGIS system has frequently been validated^26–28,33^ and used successfully in several epidemiological studies.^34–36^ The model has been shown to have high geographical and temporal predictive validity. For example, comparing modelled and measured 1 month mean NO_2_ concentrations in Greater Copenhagen showed a correlation coefficient of 0.78.^28^ Correlations ranged from 0.67 to 0.86 for PM_2.5_ and from 0.76 to 0.79 for BC depending on measurement series and averaging time.^27^

The modelling process provided annual average exposure estimates of PM_2.5_, BC, O_3_ and NO_2_ at the street level. Residential address histories from 1979 forward were obtained from the Danish Civil Registration System,^15^ for cases and controls. All addresses were geocoded, and modelled estimates were assigned for each pollutant at each address. For participants with air pollution exposure estimates for 80% or more of the 10 years prior to the diagnosis date (index date for controls), a time-weighted average was determined for each pollutant exposure over all residential addresses during the time period. The most relevant exposure time window for environmental exposures and incident leukaemia is currently under debate,^37–39^ thus we also considered exposures averaged over 1 and 5 years prior to diagnosis.

### Statistical analyses

Descriptive statistics were computed for individual and area-level socioeconomic position variables, and we calculated Spearman correlation coefficients between pollutants. We conducted conditional logistic regression with separate models for exposures averaged over 1, 5 and 10 years prior to diagnosis (or index date) for each pollutant beginning with a basic model (age, sex and calendar time matching only) and followed by adjustment for individual-level covariates: occupation (unemployed, retired and low, medium and high-skilled), marital status (married/cohabiting, divorced and never married/single), and disposable income (in quartiles). Our fully adjusted final models additionally adjusted for residential parish-level socioeconomic position indicators: percentages of population with basic education, with income in the lowest quartile, owning their own home and retired. The relationships among ambient air pollution exposures and subtypes of leukaemia (acute and chronic lymphoblastic and acute and chronic myeloid) were examined in separate models. Due to prior literature focusing on sex differences with respect to air pollution-related health outcomes, we examined effect modification by sex.^40–43^ We also examined effect modification by age group (age 70 years and older compared to younger) and whether the exclusion of participants with cardiovascular disease or inclusion of cases with topography codes other than C42.1 and their controls influenced the results. All analyses were conducted using SAS 9.3 (SAS Institute, Cary, NC, USA).

## Results

We identified 16,732 cases of leukaemia occurring since 1989 among adult residents of Denmark aged 20 years or more and 66,924 matched controls (for two cases less than four eligible controls were available). Excluding non-residents of Denmark at index date (13 cases and 4522 controls); individuals with <80% geocodable address history for the ten years prior to index date (457 cases and 2540 controls); those missing information on the covariates of interest (22 cases and 132 controls), cases with non-specific leukaemia and ill-defined topography and their controls (707 cases and 2544 controls), cases and controls of non-Danish origin (530 cases and 2194 controls), and controls missing cases and vice versa due to earlier deletions resulted in a final sample size of 14,986 cases and 51,624 controls.

The average age of cases was 67.9 years (standard deviation (SD): 14.1) and 68.7 years (SD: 13.7) for controls, with about 24% of cancers diagnosed under age 60. As shown in Table 1, ~42% of controls and cases were female. Other individuals and parish-level socio-economic statistics were similar, with the majority of participants retired (67%), married/cohabiting (59%), and living in parishes with a majority of the population owning their own home (64%) and a minority having only a basic level of education (29%). Ten-year-averaged pollutant exposures were also comparable among cases and controls with a mean exposure of ~18 µg/m^3^ for PM_2.5_, 0.8 µg/m^3^ for BC, 21 µg/m^3^ for NO_2,_ and 60 µg/m^3^ for O_3._ 1- and 5-year averaged pollutant exposures were similar (Table S2). Among 10-year-averaged pollutant exposures, BC, typically a locally emitted constituent of PM2.5 mainly produced by combustion processes (e.g. traffic, wood stoves) or forest fires, was highly correlated with NO_2_ (0.93) and strongly negatively correlated with O_3_ (−0.91) (Table S3). O_3_ was strongly negatively correlated with NO_2_ (−0.98), likely reflecting that NO_2_ is produced from NO and O_3_ during the day and the reverse occurs at night. Correlations were similar for shorter time-averaging periods. The correlation between the same pollutant exposure over different time-averaging periods was high for all pollutants (>0.90).

**Table 1:** Descriptive characteristics by case and control status.

	Controls^a^	Cases
	(*N* = 51,624)	(*N* = 14,986)
	% (*N*)	% (*N*)
Individual level variables		
Female	42.4 (21,894)	41.5 (6215)
Occupation		
Unemployed	2.1 (1059)	2.3 (339)
Low skill level	13.6 (7042)	14.6 (2191)
Medium skill level	11.6 (5984)	12.3 (1840)
High skill level	5.2 (2688)	5.3 (793)
Retired	67.5 (34,851)	65.6 (9823)
Marital status		
Married/cohabiting	58.5 (30,188)	59.4 (8897)
Divorced	31.7 (16,341)	30.7 (4596)
Never married/single	9.9 (5095)	10.0 (1493)
Disposable income		
Quartile 1	24.6 (12,695)	22.8 (3414)
Quartile 2	25.1 (12,974)	25.2 (3780)
Quartile 3	25.2 (13,004)	25.8 (3867)
Quartile 4	25.1 (12,951)	26.2 (3925)

Area level variables	Mean (SD)	Mean(SD)
% population with only basic education	29.5 (10.7)	29.1 (10.5)
% population in 1st income quartile	10.8 (5.5)	10.6 (5.4)
% population home owners	64.2 (22.7)	64.6 (22.3)
% population retired	6.4 (3.2)	6.3 (3.2)
Residential pollutant exposure estimates averaged over 10 years prior to diagnosis		
PM_2.5_ (µg/m^3^)	18.1 (3.6)	18.0 (3.6)
BC (µg/m^3^)	0.8 (0.5)	0.8 (0.5)
NO_2_ (µg/m^3^)	21.0 (8.5)	20.8 (8.5)
O_3_ (µg/m^3^)	59.7 (7.6)	59.9 (7.6)

^a^Controls matched to cases by sex, and birth month and year.

Results from basic, individual-level and fully adjusted conditional logistic regression models are shown for a 10 µg/m^3^ unit increase in estimated exposures averaged over 1, 5 and 10 years prior to diagnosis for PM_2.5_, NO_2_, and O_3_ and a 1 µg/m^3^ increase in estimated BC exposure (Table 2a). Adjustment for potential confounders did not appreciably change results. Fully adjusted models showed that PM_2.5_ exposure averaged over the 10 years prior to diagnosis was associated with increased odds for leukaemia (odds ratio (OR): 1.17, 95% confidence interval (CI): 1.04, 1.32) and the OR for BC was 1.04 (95% CI: 0.99, 1.08). Given that the most relevant time period of environmental exposures for incident leukaemia has not yet been established, we also examined air pollutant exposures averaged over 1- and 5-year periods prior to diagnosis. We observed stronger associations for PM_2.5_ (1-year mean exposure OR: 1.24, 95% CI: 1.09, 1.41) and BC (1-year mean exposure OR: 1.06, 95% CI: 1.01,1.11) compared to these associations with longer averaging periods (5 and 10 years) (Table 2a). Results from fully adjusted models using an IQR increase in pollutant exposure are comparable (Table S4). We tested deviation from linearity by comparing a decile model with a linear model using the likelihood ratio test and found no deviation (all *p* > 0.141, Supplemental Table S5).

**Table 2:** (a) Odds ratios for the associations of all leukaemias with residential air pollution exposures 1, 5 and 10 years prior to diagnosis; (b) odds ratios for the associations of all leukaemias with residential air pollution exposures 1, 5 and 10 years prior to diagnosis; age stratified analysis; age up to 69 (cases = 7246 controls = 23,676); (c) odds ratios for the associations of all leukaemias with residential air pollution exposures 1, 5 and 10 years prior to diagnosis; age stratified analysis; age group 70 and above (cases = 7740 controls = 27,948).

Pollutant	Time-weighted	Basic model^a^	Individual level^b^	Area level^c^
Exposures	Average	OR (95% CI)	OR (95% CI)	OR (95% CI)
(a) PM_2.5_ (10 µg/m^3^)	1 year	1.19 (1.06–1.34)	1.19 (1.06–1.34)	1.24 (1.09–1.41)
	5 years	1.15 (1.02–1.29)	1.15 (1.02–1.29)	1.19 (1.05–1.35)
	10 years	1.13 (1.01–1.26)	1.13 (1.01–1.26)	1.17 (1.03–1.32)
BC (1 µg/m^3^)	1 year	1.04 (1.00–1.08)	1.04 (1.00–1.08)	1.06 (1.01–1.11)
	5 years	1.03 (0.99–1.07)	1.03 (0.99–1.07)	1.05 (1.01–1.10)
	10 years	1.02 (0.98–1.06)	1.02 (0.98–1.06)	1.04 (0.99–1.09)
NO_2_ (10 µg/m^3^)	1 year	1.00 (0.97–1.02)	1.00 (0.97–1.02)	1.01 (0.98–1.04)
	5 years	1.00 (0.97–1.02)	1.00 (0.97–1.02)	1.01 (0.97–1.04)
	10 years	0.99 (0.97–1.01)	0.99 (0.97–1.01)	1.00 (0.97–1.03)
O_3_ (10 µg/m^3^)	1 year	1.00 (0.98–1.03)	1.00 (0.98–1.03)	0.99 (0.95–1.03)
	5 years	1.01 (0.98–1.04)	1.01 (0.98–1.04)	1.00 (0.96–1.04)
	10 years	1.01 (0.99–1.04)	1.01 (0.99–1.04)	1.01 (0.97–1.05)
(b) PM_2.5_ (10 µg/m^3^)	1 year	0.975 (0.815–1.166)	0.981 (0.820–1.175)	0.989 (0.813–1.204)
	5 years	0.944 (0.790–1.128)	0.950 (0.794–1.136)	0.955 (0.786–1.161)
	10 years	0.929 (0.781–1.105)	0.933 (0.783–1.112)	0.939 (0.778–1.134)
BC (1 µg/m^3^)	1 year	1.013 (0.955–1.074)	1.016 (0.958–1.077)	1.033 (0.968–1.102)
	5 years	1.008 (0.952–1.068)	1.011 (0.955–1.071)	1.028 (0.965–1.095)
	10 years	1.002 (0.946–1.062)	1.005 (0.948–1.065)	1.021 (0.958–1.088)
NO_2_ (10 µg/m^3^)	1 year	0.971 (0.938–1.006)	0.973 (0.940–1.009)	0.972 (0.926–1.020)
	5 years	0.974 (0.940–1.008)	0.976 (0.942–1.012)	0.977 (0.931–1.025)
	10 years	0.971 (0.937–1.005)	0.973 (0.939–1.008)	0.971 (0.925–1.018)
O_3_ (10 µg/m^3^)	1 year	1.027 (0.987–1.069)	1.024 (0.983–1.067)	1.025 (0.969–1.084)
	5 years	1.029 (0.989–1.071)	1.026 (0.985–1.069)	1.028 (0.972–1.088)
	10 years	1.032 (0.991–1.073)	1.029 (0.988–1.071)	1.033 (0.978–1.091)
(c) PM_2.5_ (10 µg/m^3^)	1 year	1.379 (1.181–1.610)	1.372 (1.174–1.603)	1.453 (1.227–1.722)
	5 years	1.318 (1.134–1.532)	1.311 (1.127–1.526)	1.379 (1.169–1.627)
	10 years	1.295 (1.119–1.498)	1.290 (1.114–1.494)	1.349 (1.151–1.582)
BC (1 µg/m^3^)	1 year	1.063 (1.007–1.123)	1.061 (1.005–1.121)	1.085 (1.018–1.155)
	5 years	1.049 (0.994–1.106)	1.047 (0.993–1.105)	1.067 (1.004–1.134)
	10 years	1.034 (0.981–1.091)	1.033 (0.979–1.090)	1.049 (0.987–1.116)
NO_2_ (10 µg/m^3^)	1 year	1.015 (0.984–1.047)	1.014 (0.983–1.046)	1.034 (0.990–1.079)
	5 years	1.011 (0.981–1.043)	1.010 (0.979–1.042)	1.027 (0.984–1.072)
	10 years	1.006 (0.976–1.037)	1.005 (0.975–1.036)	1.018 (0.975–1.062)
O_3_ (10 µg/m^3^)	1 year	0.992 (0.957–1.028)	0.986 (0.951–1.023)	0.965 (0.917–1.015)
	5 years	0.984 (0.950–1.020)	0.994 (0.959–1.031)	0.979 (0.930–1.030)
	10 years	0.998 (0.964–1.034)	1.001 (0.966–1.023)	0.991 (0.943–1.042)

^a^Basic model.

^b^Individual level model = Basic model plus occupation, marital status, and individual level disposable income (quartiles).

^c^Area level model = Individual level model plus area level education, income, home ownership, and retirement.

Analyses examining effect modification by age group showed null associations for all pollutant exposures and time periods examined for participants younger than 70 years old (Table 2b), however strong positive associations were observed in fully adjusted models for PM2.5 exposures averaged over 1, 5 and 10 years prior to diagnosis (OR for 1-year average: 1.45, 95% CI: 1.23, 1.72; 5 years average: 1.38; 95% CI: 1.17, 1.63; 10 years average: 1.35, 1.15, 1.58) and BC exposures averaged over 1 year prior to diagnosis (OR: 1.08, 95% CI: 1.02, 1.15) for participants at least 70 years of age (Table 2c). Stratification by sex did not appreciably change results nor did the exclusion of participants with pre-existing cardiovascular disease or inclusion of cases with unspecified leukaemia (topography other than C42.1) and their controls (results not shown).

In fully adjusted models stratified by four main subtypes of leukaemia, we observed positive associations with PM_2.5_ exposures averaged over the 10 years prior to the diagnosis of chronic lymphoblastic leukaemia and acute myeloid leukaemia (Table 3). Stronger relationships were observed for shorter exposure averaging time periods. Higher exposure to PM_2.5_ over the year prior to diagnosis was associated with higher odds of chronic lymphoblastic (OR: 1.23, 95% CI: 1.03,1.48) and acute myeloid (OR: 1.31, 95% CI: 1.04,1.65) leukaemias (Table 3). Relationships between BC and chronic lymphoblastic and acute myeloid leukaemias showed similar patterns.

**Table 3:** Odds ratios from fully adjusted models^a^ for the associations of all lymphoblastic and myeloid leukaemias with traffic-related residential estimated air pollution exposures for 1, 5 and 10 years prior to diagnosis or index date.

Pollutant exposures	Time-weighted average	Acute lymphoblastic	Chronic lymphoblastic	Acute myeloid	Chronic myeloid
		Cases = 547	Cases = 7402	Cases = 4220	Cases = 1433
		OR (95% CI)	OR (95% CI)	OR (95% CI)	OR (95% CI)
PM_2.5_ (10 µg/m^3^)	1 year	0.78 (0.38–1.62)	1.23 (1.03–1.48)	1.31 (1.04–1.65)	1.10 (0.74–1.65)
	5 years	0.86 (0.41–1.81)	1.20 (1.00–1.43)	1.21 (0.96–1.52)	1.03 (0.69–1.54)
	10 years	0.91 (0.44–1.89)	1.15 (0.97–1.37)	1.22 (0.98–1.53)	0.96 (0.65–1.42)
BC (1 µg/m^3^)	1 year	0.96 (0.74–1.26)	1.07 (1.00–1.14)	1.08 (1.00–1.17)	1.00 (0.84– 1.18)
	5 years	1.01 (0.77–1.31)	1.05 (0.99–1.12)	1.07 (0.99–1.16)	0.98 (0.83– 1.16)
	10 years	1.06 (0.81–1.38)	1.03 (0.97–1.10)	1.06 (0.98–1.15)	0.95 (0.80–1.13)
NO_2_ (10 µg/m^3^)	1 year	0.96 (0.80–1.15)	1.00 (0.96–1.05)	1.02 (0.97–1.09)	0.99 (0.89–1.09)
	5 years	1.03 (0.87–1.24)	1.00 (0.96–1.05)	1.02 (0.96–1.08)	0.98 (0.89–1.09)
	10 years	1.06 (0.89–1.26)	0.99 (0.95–1.04)	1.01 (0.95–1.07)	0.96 (0.86–1.06)
O_3_ (10 µg/m^3^)	1 year	1.04 (0.84–1.28)	1.01 (0.96–1.06)	0.96 (0.90– 1.03)	1.02 (0.90–1.14)
	5 years	0.95 (0.77–1.17)	1.01 (0.96–1.06)	0.98 (0.92– 1.05)	1.05 (0.93–1.18)
	10 years	0.91 (0.75–1.12)	1.02 (0.97–1.08)	0.99 (0.93–1.06)	1.08 (0.96–1.22)

^a^Adjusting for individual-level occupation, marital status, and disposable income (quartiles); and area-level education, income, home ownership and retirement.

## Discussion

In this nationwide population-based case-control study, we observed a positive relationship between incident leukaemia and increased long-term exposure to ambient PM_2.5_ averaged over 1, 5 and 10 years prior to diagnosis. We also observed a positive relationship between BC exposures averaged 1 year prior to diagnosis and incident leukaemia. Age stratified analyses showed that these associations were driven by relationships among the study population that was 70 years of age and older, compared to null findings among participants younger than 70 years of age. In this population, we did not observe associations of incident leukaemia with long-term exposure to NO_2_ or O_3_ for any of the time windows examined. In models stratified by subtype of leukaemia, estimates of similar size to those found for all incident leukaemias were found for incident chronic lymphoblastic and acute myeloid leukaemia with PM_2.5_ exposures averaged over 5 and 10 years prior to diagnosis. However, we observed higher estimates for these subtypes with PM_2.5_ exposures averaged over the year prior to diagnosis.

Comparison of our findings with those of prior studies of air pollution exposures and leukaemia are challenging due to the scarcity of literature on the topic. In one of the few prior studies on adult leukaemia and ambient air pollution exposures, Raaschou-Nielsen et al.^14^ also used the Danish Cancer Registry to identify 1967 cases and matched them with 3881 controls. Similar to our findings, the authors did not observe any strong associations between NO_2_ exposures averaged over 5 years or 10 years with myeloid or lymphocytic leukaemia. However, with longer NO_2_ exposures (20 years), estimated from 1971, they found increases in incident acute myeloid leukaemia and reported findings suggestive of higher ORs among older participants. Differences between our studies could be due to NO_2_ exposures generally decreasing over time in Denmark.^44^ Our overall results for long-term NO_2_ exposures with lymphoblastic leukaemias are comparable with the null findings observed by Winters et al.^13^ for a Canadian case-control study of 1066 leukaemia cases and 5,039 controls. However, the authors reported evidence of a weak association at low exposures for total leukaemias. In contrast to the positive relationship we observed with PM_2.5_ exposures and total leukaemias among older participants and for chronic lymphoblastic leukaemias, Winters et al.^13^ did not report any consistently strong positive associations. However, the exposure levels were lower in the Canadian study (mean 18.0 vs 11.7 µg/m^3^), age-stratified results were not presented, and the exposure was estimated over a 20-year period prior to diagnosis. These are possible explanations for the different findings as our data suggest that the most relevant exposure window may be only a few years before diagnosis and among older age groups, and it may be difficult to discern associations with longer time averaging periods overall.

Few other studies have examined adult leukaemia and ambient air pollution with a mixture of exposure assessment methods and outcomes. A case-control study conducted in Northern Italy between 2002 and 2005 did not find any relationships between leukaemia and zones of air pollution exposure categorised by level of industrial activity, urbanicity and wind direction.^45^ The authors reported that study limitations included the small sample size and limited availability of air pollution monitoring data. An Iranian study of leukaemia mortality found that rates correlated strongly with NO_2_ and carbon monoxide (CO) but not with PM_2.5_ or O_3_.^46^

More research has been conducted on childhood leukaemia as well as on occupational exposures for adult leukaemia. Findings regarding childhood leukaemia and ambient air pollution have been somewhat equivocal but suggestive, with stronger evidence with respect to ambient exposures to benzene.^12^ However, clinical treatment research as well as recent genetic research suggest that adult and childhood leukaemia, particularly certain subtypes, may differ as much as different cancers.^47,48^ Therefore, comparisons between air pollution exposure and leukaemia studies between children and adults should be made with caution.

A few recent studies have investigated potential underlying mechanisms with results lending biological plausibility for a link between leukaemia and ambient PM_2.5_ exposures. Jin et al.^49^ conducted in vitro and in vivo studies with human myeloid leukaemia cells exposed to PM_2.5_ samples collected at an urban background site in China in 2013. The authors reported PM_2.5_ exposures enhanced leukaemia cell growth and release of inflammatory cytokines. Chen et al.^50^ conducted in vivo and in vitro studies using human acute myeloid leukaemia cells. The authors observed that low doses of PM_2.5_ promoted cell growth, high doses induced cytotoxicity, and in vivo exposures for 12 weeks increased the release of inflammatory cytokines with 6 weeks of exposure.

This study has a number of strengths and weaknesses. Study strengths included capturing all cancers nationwide for 25 years, which provided a large sample size and long period of time enabling us to examine the relationship of several subtypes of leukaemias with long-term exposures averaged from 1 year to 10 years. Denmark affords residents universal access to health care coverage and has a more racial and ethnically homogeneous population than some other countries, which may somewhat limit generalisability, however, potential confounding due to variations in access to healthcare is limited. We were unable to account for potential confounding by familial leukaemia/lymphoma history, exposure to tobacco smoke or occupational exposures; but we were able to adjust for occupational skill-level and status as well as a number of individual and area-level socioeconomic position factors. Some bias may result from the exclusion of participants due to missing data, however, demographic characteristics for cases and controls in the study sample and final analysis are comparable and the proportion of excluded is low (Table S6). Like most large, population-based air pollution epidemiological studies, we were unable to assess air pollution exposures at additional locations (i.e. places of employment), nor can we completely exclude the possibility that these findings are related to air toxics that have been classified as human carcinogens and are present in air pollution. Though we used exposures estimated by a modelling system with high spatial and temporal validity,^27,29,32^ exposure misclassification will inevitably have occurred. Any resulting bias would likely be toward the null because the misclassification would be similar for addresses of cases and controls, i.e. non-differential. We also cannot rule out that findings were due to chance although the large size of our study population is a deterrent. The strengths of our exposure assessment procedures included the use of a spatio-temporal dispersion modelling setup that captured regional, background and street-level variation in air pollutant distributions, and obtaining residential histories for each individual allowed us to develop time weighted averages that included each residential address of each participant during the 10-year period.

## Conclusion

We observed that chronic PM_2.5_ exposures were associated with incident total leukaemia among older adults. The relationship appeared to be driven by associations with chronic lymphoblastic and acute myeloid leukaemias. Stronger associations were observed with shorter averaging times before diagnosis, thus interventions to reduce PM_2.5_ exposures may be associated with beneficial impacts on incident leukaemia over relatively short periods of time.

## Supplementary information

Supplementary Tables_Clean

## Data Availability

Data are located at the secure IT environment at Statistics Denmark. Access to the data of Statistics Denmark can be obtained if approved by Statics Denmark. Requirements include compliance with the EU General Data Protection Regulation and the Danish Data Protection Act.
