# Supplementary material for: Relationship of leukaemias with long-term ambient air pollution exposures in the adult Danish population
Source: Br J Cancer. 2020 Sep 17;123(12):1818–24. doi: 10.1038/s41416-020-01058-2 (PMC7722932; doi:10.1038/s41416-020-01058-2)
Supplement: Supplementary file 1 — Supplementary Tables_Clean [file 41416_2020_1058_MOESM1_ESM.docx]

|  | **Cases** | **Controls** |
| --- | --- | --- |
| **All Leukemia Diagnoses** | 22108 | 88428 |
| **Exclusion Reason** |  |  |
| - Diagnosed prior to 1989 | 5376 | 21504 |
| - Greenland | 6 | 55 |
| - Emigrate before index | 5 | 42 |
| - Miss emigrate before index | 0 | 3797 |
| - Disappear before index | 0 | 217 |
| - Miss Tax code | 2 | 0 |
| - Non-residents of Denmark | 681 | 2769 |
| - non-specific ill-defined topography | 719 | 0 |
| - Exposure missing over 20% of time | 302 | 1647 |
| - Missing Covariates | 14 | 87 |
| - No matched case or controls | 17 | 6686 |
| **Total included in final analyses** | 14986 | 51624 |

**Supplementary Table S1. Sample selection**

**Supplementary Table S2. Residential pollutant exposure estimates averaged over 1 and 5 years prior to diagnosis**

|  | **1 year** | | **5 years** | |
| --- | --- | --- | --- | --- |
|  | **Controls*** | **Cases** | **Controls*** | **Cases** |
| PM2.5 (µg/m3) | 16.35 (3.50) | 16.25 (3.53) | 17.04 (3.48) | 16.93 (3.50) |
| BC (µg/m3) | 0.77 (0.44) | 0.77 (0.56) | 0.80 (0.45) | 0.80 (0.54) |
| NO2 (µg/m3) | 19.21 (8.69) | 19.01 (8.68) | 20.24 (8.62) | 20.04 (8.63) |
| O3 (µg/m3) | 59.68 (7.61) | 62.75 (7.98) | 60.56 (7.64) | 60.75 (7.65) |

**Supplementary Table S3**

**Spearman correlations among pollutant exposures averaged over 1, 5 and 10 years prior to diagnosis for cases and index date for controls**

|  |  | **1 year** | | | | **5 year** | | | | **10 year** | | | |
| --- | --- | --- | --- | --- | --- | --- | --- | --- | --- | --- | --- | --- | --- |
|  |  | **BC** | **PM2.5** | **NO2** | **O3** | **BC** | **PM2.5** | **NO2** | **O3** | **BC** | **PM2.5** | **NO2** | **O3** |
| **1 year** | | | | | | | | | | | | | |
|  | BC | 1 |  |  |  |  |  |  |  |  |  |  |  |
|  | PM2.5 | 0.57 | 1 |  |  |  |  |  |  |  |  |  |  |
|  | NO2 | 0.91 | 0.61 | 1 |  |  |  |  |  |  |  |  |  |
|  | O3 | -0.86 | -0.57 | -0.93 | 1 |  |  |  |  |  |  |  |  |
| **5 year** | | | | | | | | | | | | | |
|  | BC | 0.94 | 0.50 | 0.90 | -0.83 | 1 |  |  |  |  |  |  |  |
|  | PM2.5 | 0.54 | 0.96 | 0.61 | -0.57 | 0.53 | 1 |  |  |  |  |  |  |
|  | NO2 | 0.88 | 0.56 | 0.97 | -09 | 0.92 | 0.59 | 1 |  |  |  |  |  |
|  | O3 | -0.85 | -0.58 | -0.95 | 0.93 | -0.9 | -0.61 | -0.97 | 1 |  |  |  |  |
| **10 year** | | | | | | | | | | | | | |
|  | BC | 0.91 | 0.47 | 0.87 | -0.81 | 0.98 | 0.49 | 0.91 | -0.89 | 1 |  |  |  |
|  | PM2.5 | 0.52 | 0.95 | 0.60 | -0.57 | 0.52 | 0.99 | -0.59 | -0.61 | 0.49 | 1 |  |  |
|  | NO2 | 0.85 | 0.51 | 0.95 | -0.87 | 0.91 | 0.54 | 0.98 | -0.96 | 0.93 | 0.54 | 1 |  |
|  | O3 | -0.84 | -0.55 | -0.94 | 0.91 | -0.89 | -0.58 | -0.97 | 0.98 | -0.91 | -0.58 | -0.98 | 1 |

| **Pollutant Exposures (IQR)*** | **TWA** | **Area Level**** |
| --- | --- | --- |
|  |  | **OR (95% CI)** |
| PM_2.5_ (5.58 µg/m^3)^ | 1 | 1.127 (1.050 – 1.211) |
|  | 5 | 1.102 (1.028 – 1.182) |
|  | 10 | 1.092 (1.020 – 1.167) |
| BC (0.394 µg/m^3^) | 1 | 1.025 (1.006 – 1.044) |
|  | 5 | 1.020 (1.002 – 1.039) |
|  | 10 | 1.015 (0.997 – 1.034) |
| NO_2_ (11.0 µg/m^3^) | 1 | 1.007 (0.973 – 1.041) |
|  | 5 | 1.006 (0.972 – 1.040) |
|  | 10 | 0.998 (0.964 – 1.031) |
| O_3_ (9.92 µg/m^3^) | 1 | 0.992 (0.957 – 1.028) |
|  | 5 | 1.000 (0.965 – 1.040) |
|  | 10 | 1.009 (0.973 – 1.045) |

**Supplementary Table S4. Odds ratios for the associations of all leukemia with residential air pollution exposures 1, 5 and 10 years prior to diagnosis**

*IQR calculated for controls 10-year exposure

** adjusted for sex, age and calendar time, by matched design additionally adjusted for occupation, marital status, and individual level disposable income (quartiles), area level education, income, home ownership, and retirement

**Supplementary Table S5**. Check for Linearity

| **Pollutant Exposures** | P value ^1^ |
| --- | --- |
| PM_2.5_ (10 µg/m^3^) | 0.175 |
| BC (1 µg/m^3^) | 0.615 |
| NO_2_ (10 µg/m^3^) | 0.471 |
| O_3_ (10 µg/m^3^) | 0.141 |

^1^ Fully adjusted linear model compared with fully adjusted decile model

| **Supplementary Table S6. Individual level socio-demographic characteristics**  **of study participants and those excluded** | | | | | | |  |
| --- | --- | --- | --- | --- | --- | --- | --- |
|  | **Study Sample** | | **Final Analysis** | | **Excluded** | | |
|  | **Case** | **Control** | **Case** | **Control** | **Case** | **Control** | |
| **Characteristics** | 15,309 | 54,392 | 14,986 | 51,624 | 323 | 2768 | |
| **Gender** |  |  |  |  |  |  | |
| Male | 58,7% | 58,1% | 58,5% | 57,6% | 66,9% | 67,7% | |
| Female | 41,3% | 41,9% | 41,5% | 42,4% | 33,1% | 32,3% | |
| **Age (years)** |  |  |  |  |  |  | |
| <40 | 5,2% | 4,7% | 4,9% | 4,2% | 18,6% | 12,9% | |
| 40-50 | 6,8% | 6,2% | 6,7% | 5,9% | 11,1% | 11,6% | |
| 50-60 | 14,4% | 13,7% | 14,3% | 13,4% | 16,4% | 18,6% | |
| 60> | 73,6% | 75,4% | 74,1% | 76,4% | 53,9% | 57,0% | |
| **Year of leukaemia diagnosis ^1^** |  |  |  |  |  |  | |
| 1989-1993 | 18,5% | 19,7% | 18,4% | 19,4% | 27,6% | 24,5% | |
| 1994-1998 | 19,4% | 20,2% | 19,5% | 20,4% | 14,6% | 17,7% | |
| 1999-2003 | 20,8% | 20,9% | 20,8% | 20,9% | 22,0% | 21,4% | |
| 2004-2008 | 18,5% | 18,1% | 18,5% | 18,1% | 17,0% | 17,7% | |
| 2009-2014 | 22,8% | 21,1% | 22,8% | 21,2% | 18,9% | 18,7% | |
| **Disposable Income** |  |  |  |  |  |  | |
| Quintiles 1 (low) | 18,9% | 20,3% | 19,1% | 20,3% | 20,7% | 18,8% | |
| Quintiles 2 | 19,9% | 20,0% | 19,7% | 20,1% | 17,0% | 18,9% | |
| Quintiles 3 | 19,9% | 19,9% | 19,9% | 20,0% | 18,3% | 18,8% | |
| Quintiles 4 | 20,6% | 19,8% | 20,7% | 19,8% | 20,7% | 18,4% | |
| Quintiles 5 (high) | 20,6% | 19,8% | 20,7% | 19,8% | 16,1% | 18,7% | |
| Missing | 0,2% | 0,3% | 0,0% | 0,0% | 7,1% | 6,5% | |
| **Marital status** |  |  |  |  |  |  | |
| Cohabiting | 59,0% | 57,7% | 59,4% | 58,5% | 39,9% | 43,5% | |
| Divorced | 30,5% | 31,1% | 30,7% | 31,7% | 22,0% | 21,7% | |
| Never Married | 10,1% | 10,1% | 10,0% | 9,9% | 16,4% | 14,1% | |
| Missing | 0,5% | 1,1% | 0,0% | 0,0% | 21,7% | 20,7% | |
| **Attachment to labor market** |  |  |  |  |  |  | |
| Blue collar | 14,7% | 13,7% | 14,6% | 13,6% | 18,0% | 14,9% | |
| Lower white collar | 12,3% | 11,7% | 12,3% | 11,6% | 11,1% | 13,0% | |
| Higher white collar | 5,3% | 5,4% | 5,3% | 5,2% | 7,1% | 8,4% | |
| Unemployed | 2,3% | 2,2% | 2,3% | 2,1% | 3,7% | 4,8% | |
| Retired | 65,3% | 66,8% | 65,5% | 67,5% | 55,1% | 53,3% | |
| Missing | 0,1% | 0,3% | % | % | 5,0% | 5,6% | |

^1^ For controls year of index date
